# An improved Deeplab V3+ network based coconut CT image segmentation method

**DOI:** 10.3389/fpls.2023.1139666

**Published:** 2023-12-08

**Authors:** Qianfan Liu, Yu Zhang, Jing Chen, Chengxu Sun, Mengxing Huang, Mingwei Che, Chun Li, Shenghuang Lin

**Affiliations:** ^1^ School of Computer Science and Technology, Hainan University, Haikou, China; ^2^ Central South University Xiangya School of Medicine Affiliated Haikou Hospital, Haikou, China; ^3^ Coconut Research Institute, Chinese Academy of Tropical Agricultural Sciences, Wenchang, Hainan, China; ^4^ School of Information and Communication Engineering, Hainan University, Haikou, China

**Keywords:** coconut, CT images, semantic segmentation, DASPP, CBAM, RRM

## Abstract

Due to the unique structure of coconuts, their cultivation heavily relies on manual experience, making it difficult to accurately and timely observe their internal characteristics. This limitation severely hinders the optimization of coconut breeding. To address this issue, we propose a new model based on the improved architecture of Deeplab V3+. We replace the original ASPP(Atrous Spatial Pyramid Pooling) structure with a dense atrous spatial pyramid pooling module and introduce CBAM(Convolutional Block Attention Module). This approach resolves the issue of information loss due to sparse sampling and effectively captures global features. Additionally, we embed a RRM(residual refinement module) after the output level of the decoder to optimize boundary information between organs. Multiple model comparisons and ablation experiments are conducted, demonstrating that the improved segmentation algorithm achieves higher accuracy when dealing with diverse coconut organ CT(Computed Tomography) images. Our work provides a new solution for accurately segmenting internal coconut organs, which facilitates scientific decision-making for coconut researchers at different stages of growth.

## 1 Introduction

As a plant native to tropical environments, coconuts not only serve as distinctive landscape trees for tourism, but also contribute significantly to the local economy as a pillar industry. The various structures within coconuts are essential materials in other industries and closely linked to people’s lives (Arumugam and Hatta, 2022). As a result, the development of the coconut industry has garnered high attention and research efforts worldwide. However, the unique growth environment of coconuts, coupled with factors such as extensive farming practices, limited processing enterprises, weak risk resilience, low technological content, and backward deep processing capabilities, have led to insufficient raw materials and severe homogeneity issues in coconut products. Currently, the global coconut market is facing a severe supply-demand imbalance, with a significant shortage of high-quality coconuts. Consequently, the cultivation of superior coconut seeds has become a research hotspot in order to provide higher-quality seedlings and resources for the coconut industry. Real-time monitoring of the internal structural growth during the cultivation process has become the key to addressing this issue. Currently, growers can only resort to destructive methods, such as cutting open coconuts for observation and documentation, which not only hampers the normal growth of the coconut but is also unsuitable for large-scale cultivation research. However, the use of X-ray imaging methods can be effectively applied in this scenario.

Computed tomography (CT) imaging, widely used in clinical medicine, provides clear visualization of internal structures in the human body, aiding doctors in obtaining crucial information for diagnosing organs or tissues. It holds significant importance in quantitative pathological assessment, treatment planning, and disease progression monitoring. By applying this method to agricultural research, utilizing the penetrating characteristics of X-rays, we can obtain clear internal organ images of coconuts without disrupting their normal physiological structure and growth (Zhang et al., 2023).

For image segmentation tasks, traditional segmentation methods suffer from poor robustness, low efficiency, and low accuracy. With the development of deep learning techniques, image segmentation can be achieved without relying on manually designed features, as neural networks can automatically learn the features required for segmentation tasks. Therefore, methods based on deep learning have become the primary choice for researchers in various image segmentation tasks (Suk et al., 2023). However, existing deep learning-based image segmentation algorithms have significant limitations when it comes to organ segmentation tasks in coconut CT images, failing to meet the high-precision segmentation requirements in agriculture. In response to these issues, this paper proposes corresponding improvement methods and validates the effectiveness and superiority of the proposed methods through ablation experiments and comparative experiments. The model proposed in this paper can obtain higher-precision semantic information when facing coconut CT images, facilitating a more detailed analysis and evaluation of coconut development and growth.

Our work has made the following main contributions:

1. We conducted non-destructive observations of coconuts at different stages and with different characteristics through CT scanning. We obtained internal images of coconuts at multiple time periods and multiple categories. Based on the growth conditions of coconuts, we classified and labeled the internal organs of coconuts, establishing a CT-based coconut organ image dataset named “CIDCO.” These data were used for training and testing the network model we constructed and also provided image resources for coconut research.
2. To achieve precise segmentation of the internal structure of coconuts, we proposed an improved image segmentation method based on the modified Deeplab V3+ network. Through model comparison, we demonstrated that the improved network achieves higher segmentation accuracy and can be effectively applied to coconut image segmentation and growth development research.

The structure of this paper is as follows: In Section 2, we introduce and analyze relevant research on non-destructive observations and image segmentation for agricultural applications. In Section 3, we summarize the research methods used in this work. Section 4 presents the experiments we designed and compares the results with other models. In Section 5, we provide a summary of the entire work and discuss future directions and ideas.

## 2 Related work

The use of non-destructive methods to acquire images of target objects has been receiving increasing attention and gradually being applied in various research fields. CT, ultrasound, infrared laser, nuclear magnetic resonance, and other methods have been used for image scanning. For example, Yu et al. (2022) employed electron microscopy CT for non-destructive observation of coconut variations, aiming to explore growth and development. Li et al. (2020) conducted terahertz imaging to observe changes in leaf water content in their research on crop water status monitoring and diagnosis. These studies demonstrate the feasibility of obtaining images of target objects through non-destructive means. Regarding image segmentation, traditional methods include threshold determination, region-based similarity aggregation, edge operator calculations, and energy-minimizing active contour-based approaches to accomplish various segmentation tasks. For instance, Thorp and Dierig (2011) presented a color image segmentation method to monitor the flowering status of Lesquerella. This method converts the RGB color space to the HSI color space and utilizes histogram equalization to enhance image contrast. Then, threshold segmentation is used to separate the flower parts from the background, and morphological operations and region-growing algorithms are employed to remove noise and connect discontinuous flower parts. Finally, the number of flowers is counted based on the segmentation results, achieving automatic monitoring of Lesquerella flowering. Xiang (2018) introduced an image segmentation method for nighttime identification of the entire tomato plant. This method first converts the image to the HSV color space and then separates the plant from the background using threshold segmentation. However, these traditional methods perform reasonably well when dealing with images with simple linear features. But once other factors increase, they can greatly affect the segmentation results. With the rise of deep neural networks, various neural network methods have been quickly applied to various image segmentation tasks. Deep learning-based methods fundamentally transform semantic segmentation into an image per-pixel classification problem. Van De Looverbosch et al. (2021) proposed a non-destructive internal defect detection method for pears using deep learning techniques. X-ray CT scanning is employed to acquire images, and semantic segmentation techniques are used for internal defect detection and recognition. Ni et al. (2020) utilized deep learning techniques to segment and extract features from blueberry fruit images in order to better predict the harvest period and yield of blueberry fruits. This research provides a new method for accurately predicting fruit harvest and yield. Sun et al. (2021) employed semantic segmentation networks and shape-constrained level set methods to detect and segment images of apple, peach, and pear flowers. The research results demonstrate that this approach can more accurately detect and segment the contours of flowers. Turgut et al. (2022) proposed a deep learning architecture called RoseSegNet for plant organ segmentation. This model, based on attention mechanisms, can identify different organs of a rose, including petals, stamens, and leaves, providing a new tool for botanical research. Singh et al. (2022) proposed a method for semantic segmentation of cotton structures from aerial images using deep convolutional neural networks. This research achieved automatic identification and segmentation of cotton bolls from the sky using deep convolutional neural networks. This method can improve cotton harvesting efficiency, reduce costs, and provide new technological support for modern agriculture. The introduction of deep learning networks has brought faster and more accurate solutions to image segmentation tasks. However, due to the unique characteristics of coconuts, there is still limited research on the application of high-precision semantic segmentation models in coconut CT images. Therefore, our focus is on addressing this issue.

## 3 Method

### 3.1 Coconut data collection and scanning

**Figure 1:**
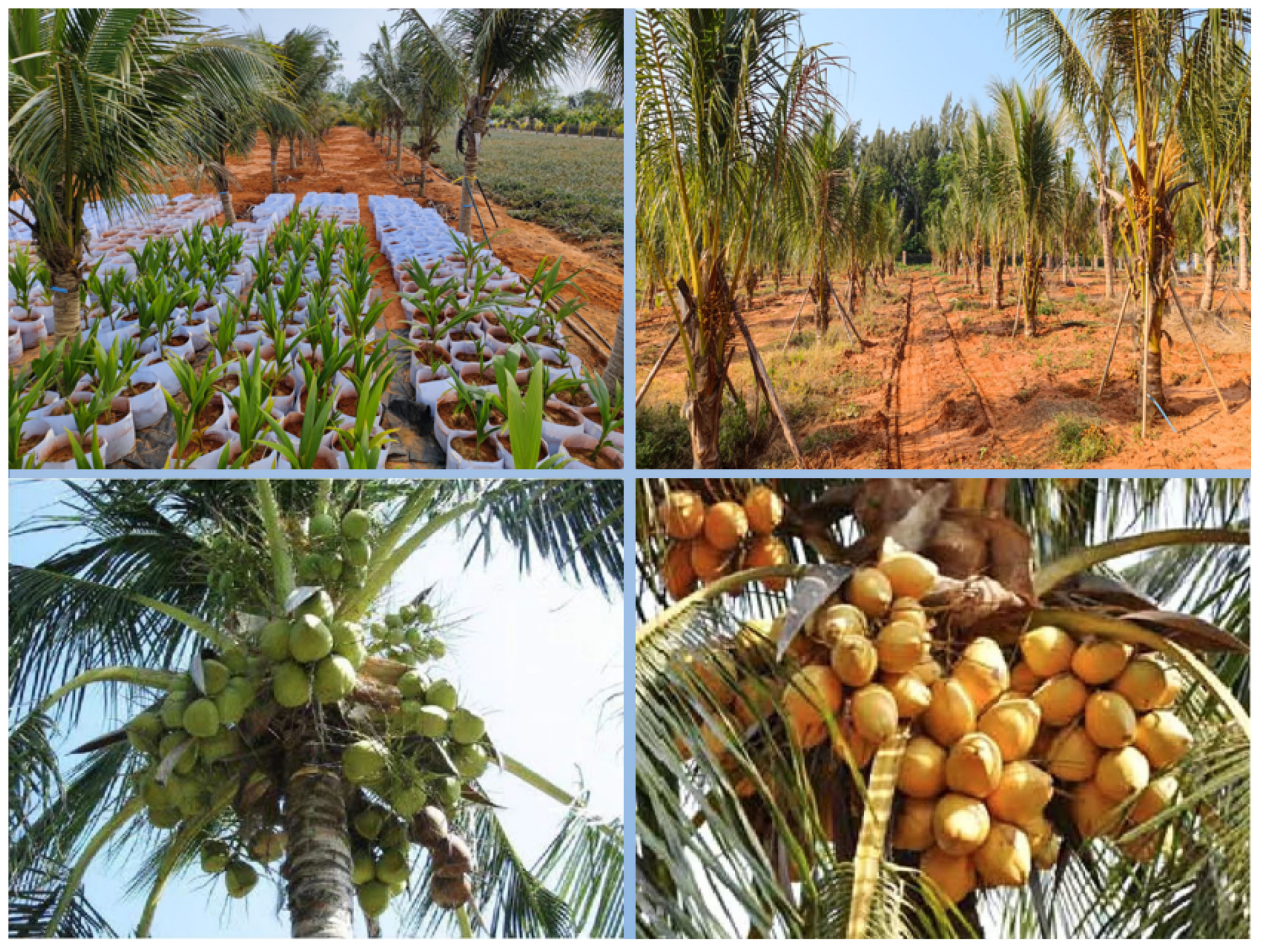
Coconut collection area situation.

Considering the suitable average temperature for coconuts to be maintained between 24 to 27°C, with ample precipitation and an annual sunlight guarantee of more than 2000 hours, and in order to obtain richer raw material resources in large-scale cultivation areas, after careful consideration, the experimental fields of Wenchang Coconut Research Institute and the coconut plantation in Leiming Town, Ding’an County were selected as the collection sites. The experimental fields adopted a triangular planting pattern to achieve higher yields per unit area, mainly consisting of green coconuts, red coconuts, and yellow coconuts, covering an age range of 3 to 12 months. The coconut trees in the plantation are approximately 20 years old, with a height of 10 meters and 30 leaves. The majority of coconuts produced are green coconuts at the stage of 7 to 12 months. Refer to **Figure 1** for illustration.

**Figure 2:**
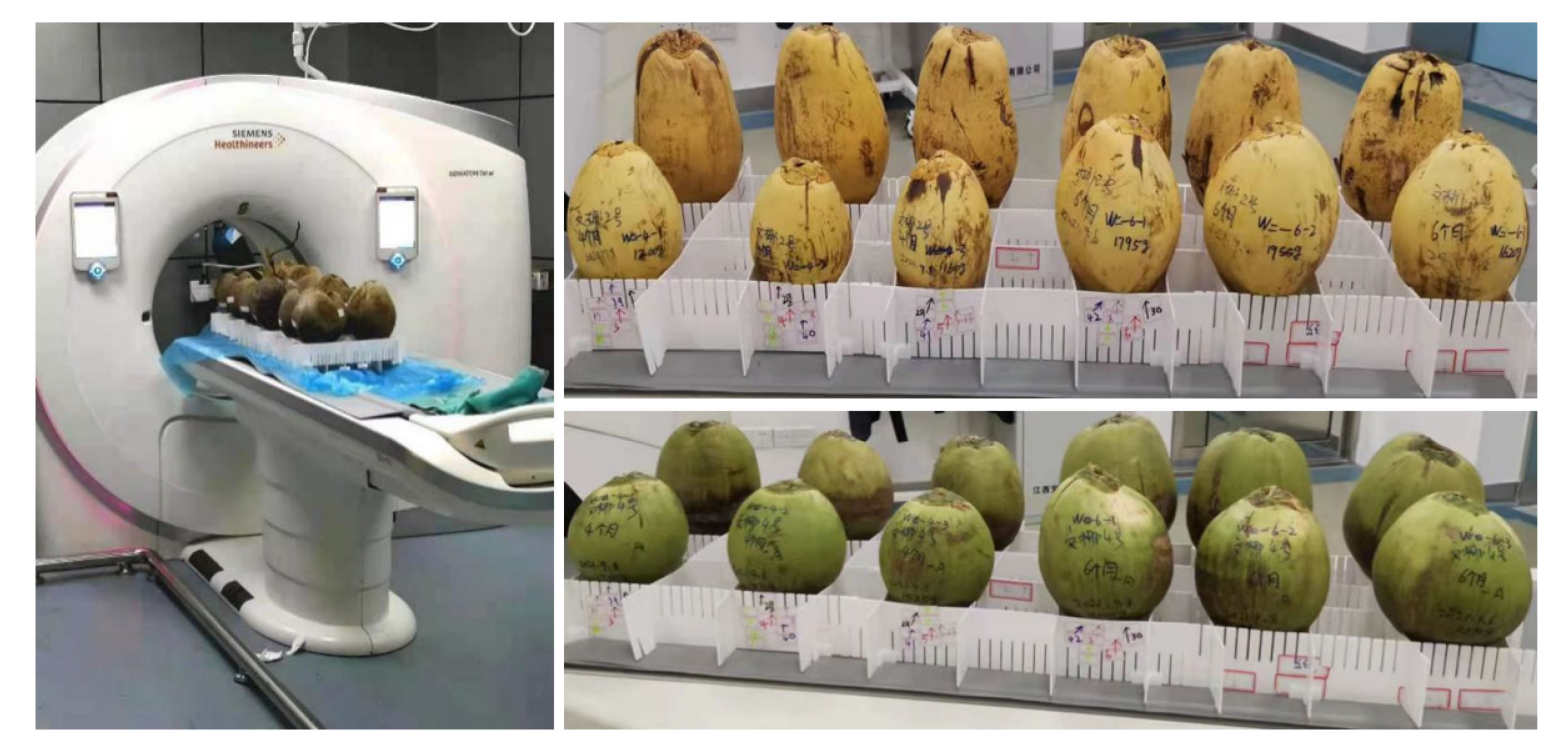
Example of coconut scan.

In the aforementioned field conditions, a total of 104 coconuts were collected, categorized into different groups based on color, type, and age. The coconuts were numbered according to their growth months in sequential order. Using the anatomical scanning of the human body as the reference position, they were scanned using a Siemens 256 dual-source CT machine. X-rays were used to obtain cross-sectional images in three directions: axial, coronal, and sagittal. This process resulted in complete multi-angle sliced images of each coconut. Considering that a single image may contain more than one complete target coconut, additional coconuts with varying representations were also included in the CT scan images. The number of images obtained from each coconut scan ranged from 170 to 220, with approximately one-fourth of the images capturing the complete structural information. An example of the coconut scanning process is shown in **Figure 2**.

**Figure 3:**
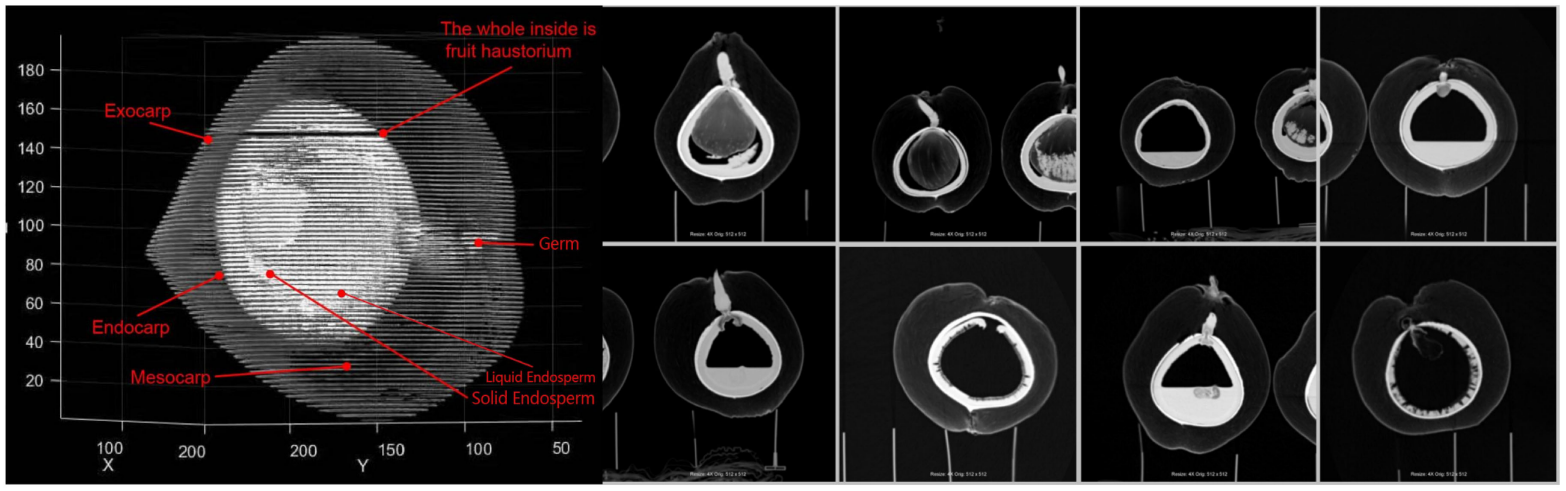
Example of CT image of coconut.

**Figure 4:**
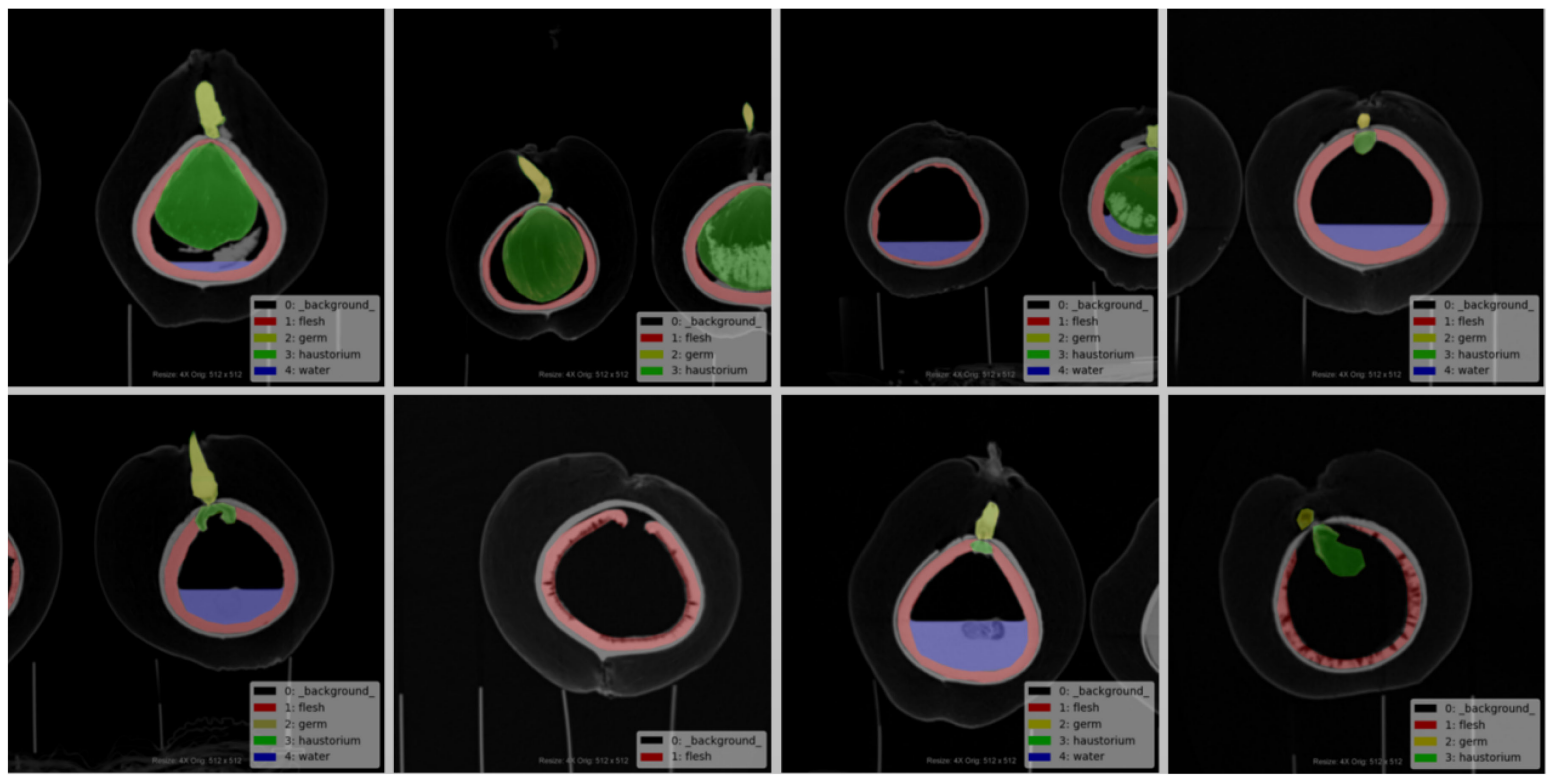
Example of the corresponding labeled diagram.

Each image is labeled in the format of “color_month_id” to facilitate quick and accurate searching. The labeled images are then stored and organized according to the major coconut varieties, with corresponding annotation folders created. Coconut researchers and project members were involved in the annotation process. The four main organs of the coconut that are most relevant to its development and growth are the absorber, solid endosperm (coconut meat), liquid endosperm (coconut water), and embryo. These four organs were annotated, with the background represented in black by default. The absorber was annotated in yellow, the solid endosperm in red, and the liquid endosperm in blue. Coconut CT images can be seen in **Figure 3**, and the corresponding annotation results are shown in **Figure 4**.

### 3.2 Design of segmentation model

Given the limitations of the original Deeplab V3+ network, such as insufficient utilization of inter-level feature information leading to unclear segmentation boundaries and lack of detailed feature map information, resulting in poor final results, we propose a new semantic segmentation model for coconut CT images. The improved model builds upon the advantages of the original framework’s encoder-decoder architecture and enhances the feature recognition and capture capabilities through module replacement and addition.

After the input of the task image, the Deeplab V3+ model first uses a deep convolutional network (DCNN) to extract features from the input image, dividing them into two categories: high-level semantic features and low-level semantic features. Some of the low-level features directly enter the decoder, while other information enters the encoder stage. At this point, the Atrous Spatial Pyramid Pooling (ASPP) module is introduced to capture coconut organ features and requires a sufficiently large receptive field. However, increasing the dilation rate leads to sparser pixel sampling compared to traditional convolution, resulting in more loss of detail information. As a result, the original ASPP module experiences attenuation in the effectiveness of dilated convolutions, and the effectiveness of atrous convolutions gradually decreases, ultimately affecting the model’s capabilities.

Furthermore, the original network employs a 4x upsampling in the decoder stage. For coconut organs, large-scale upsampling adversely affects edge segmentation. Moreover, the fusion with only low-level features from the base network may result in the loss of some information, thus affecting the final segmentation accuracy.

**Figure 5:**
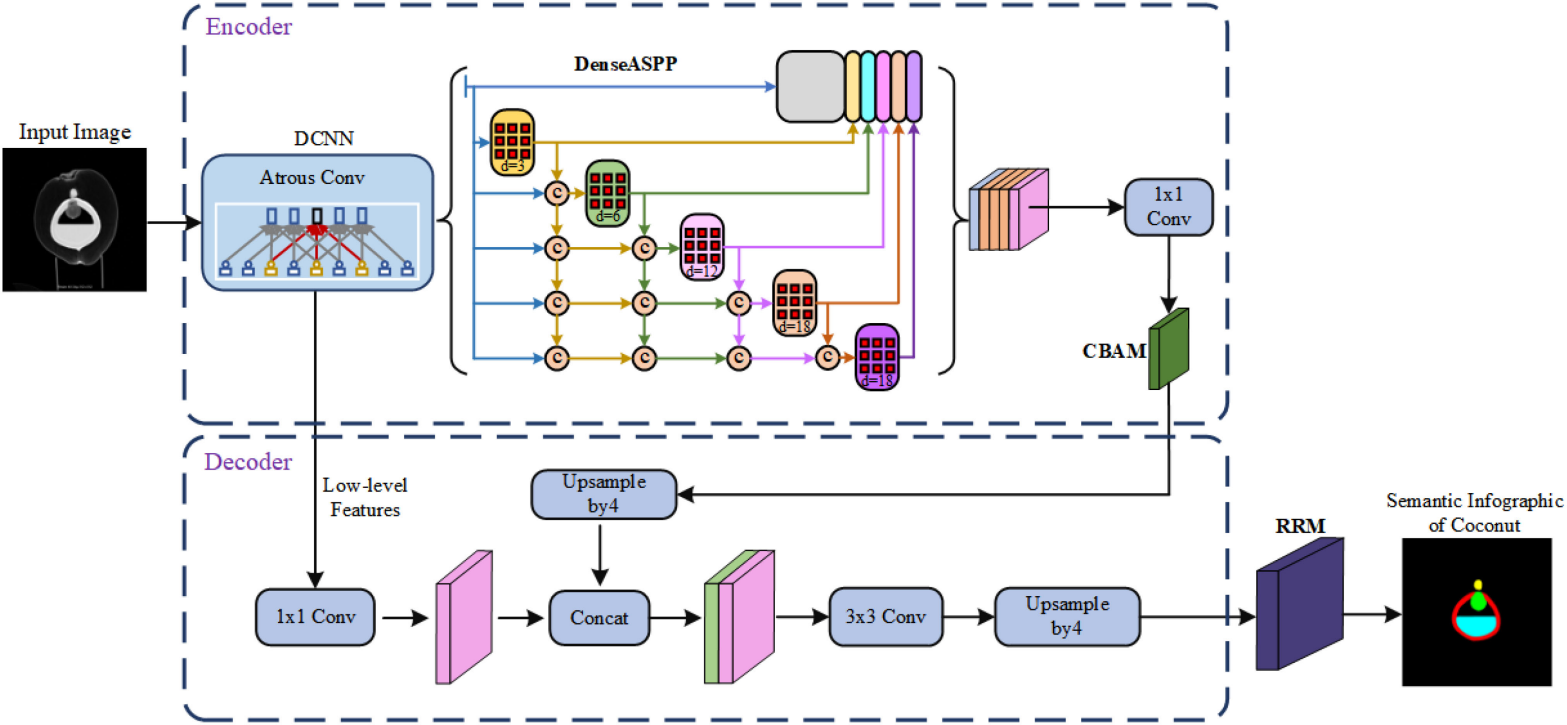
Diagram of the improved model structure.

To address these issues, the Dense Atrous Spatial Pyramid module is used to replace the original ASPP module. The input-output dense connections are established between each atrous convolution layer, allowing for the coverage of multi-scale range feature information using appropriate dilation rates. Additionally, a convolutional attention module is introduced to enhance effective feature information, suppress irrelevant information responses, and improve feature extraction and representation capabilities. Finally, a residual refinement module is embedded after the decoder to map the significant information transmitted from the upper layers, optimizing organ boundaries and improving segmentation accuracy. The improved model is illustrated in **Figure 5**.

### 3.3 Principle of the improvement module

#### 3.3.1 DASPP module

DASPP stands for “Dense Atrous Spatial Pyramid Pooling.” In the structure of the DASPP module, atrous convolutions are combined into a cascaded fusion operation. The dilation rate increases layer by layer, with layers having lower dilation rates placed in the lower-level parts and layers with higher dilation rates placed in the higher-level parts. The subsequent layers share information with the preceding layers, using their features for information sharing. This dense connectivity allows for more intensive pixel utilization. Each atrous layer concatenates the input with the output of the previous lower-level layer as its input, ultimately producing a feature map generated by multi-scale atrous convolutions.

**Figure 6:**
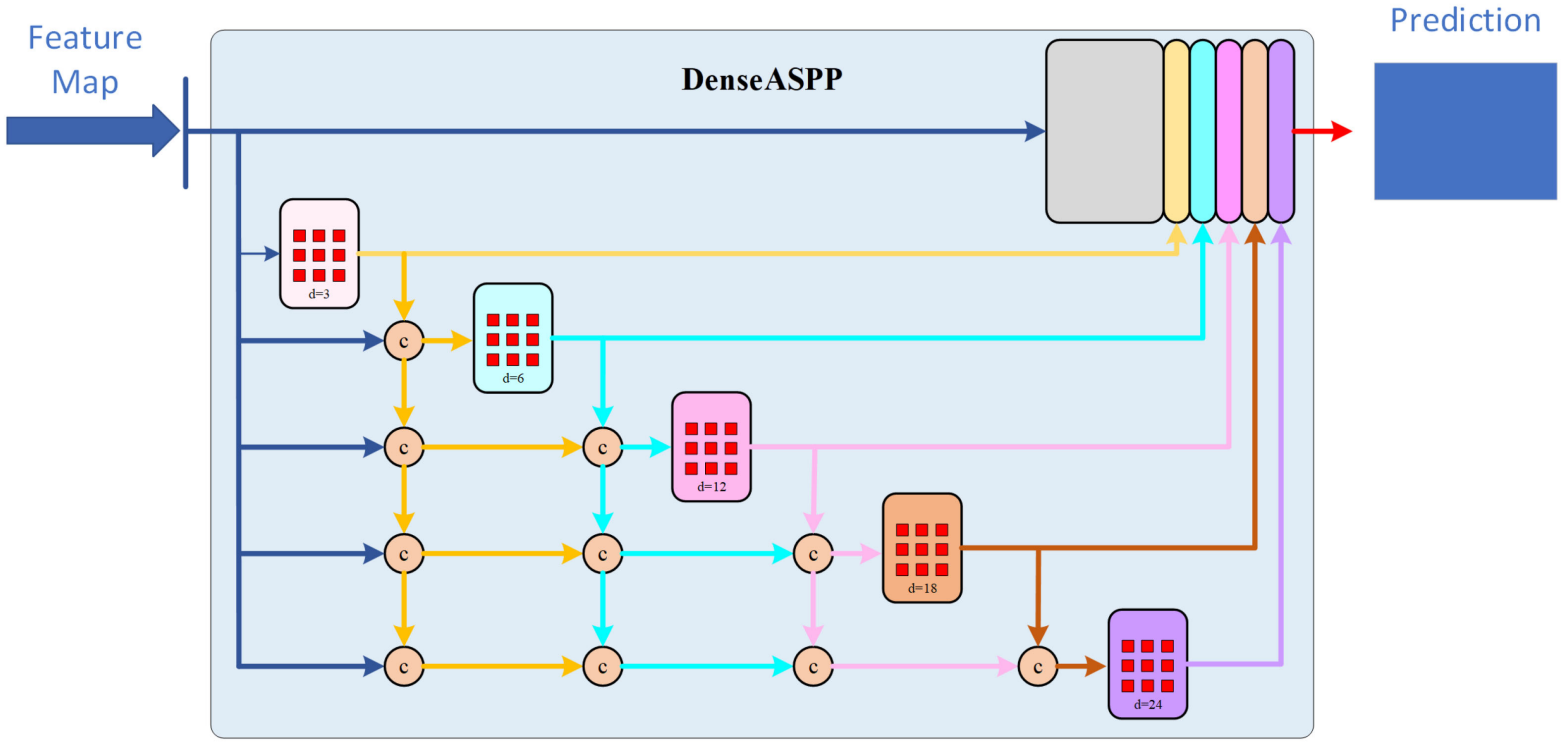
Diagram of DASPP module.

Compared to traditional ASPP, DASPP utilizes dense connections to establish interconnections between layers with different dilation rates. Each set can be considered as a convolutional kernel of a different scale, representing different receptive fields. This change brings about a denser feature pyramid and a larger receptive field, allowing for better recognition and integration of semantic features of target organs of various scales. The structure of the module is illustrated in **Figure 6**.

#### 3.3.2 CBAM attention mechanism

CBAM is a lightweight and versatile module for feed-forward convolutional neural networks. It concentrates attention resources more on the key target areas in coconut image, allocating different weights to information and background. It enhances the network’s expressive power without significantly affecting its efficiency and facilitates information propagation. CBAM consists of two main parts: Channel Attention Module and Spatial Attention Module. The input features pass through the Channel Attention Module and the Spatial Attention Module sequentially, resulting in recalibrated features.

**Figure 7:**
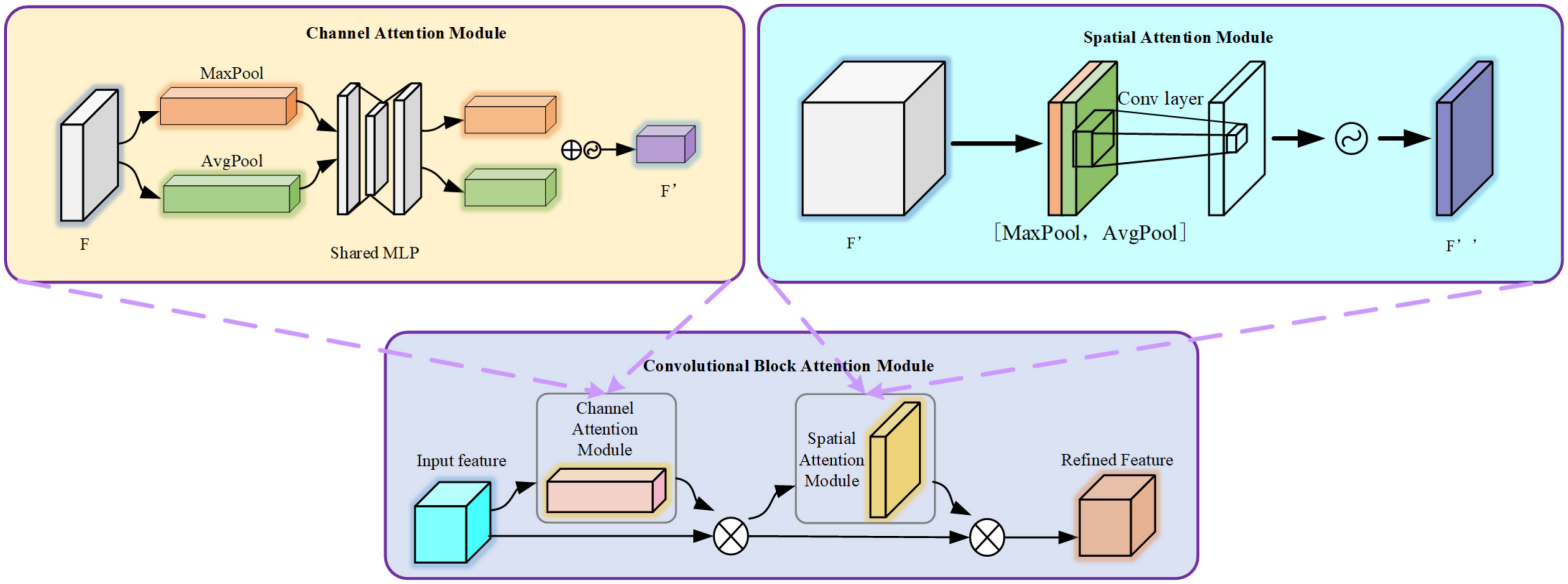
The structure of CBAM attention mechanism.

In the Channel Attention Module, both average pooling and max pooling are applied to the features. The pooled features are then fed into a shared multi-layer perceptron with shared weights. The output of the MLP is multiplied element-wise with the original feature map after a sigmoid operation. In the Spatial Attention Module, the feature map outputted by the Channel Attention Module serves as the input. Two pooling operations are performed along the channel dimension, resulting in feature maps of size h * w * 1 each time. The feature maps from the two poolings are then concatenated along the channel dimension, resulting in a feature map of size h * w * 2. This feature map undergoes a convolution operation with a kernel size of 7 * 7 and a convolutional kernel count of 1 (channel compression). The result is then passed through a sigmoid function and finally subjected to matrix multiplication. The working principle of the entire CBAM module is illustrated in **Figure 7**.

#### 3.3.3 RRM module

The Residual Refinement Module (RRM) is a commonly used module in deep neural networks that incorporates the idea of an excellent encoder-decoder architecture (Qin et al., 2019). Its main purpose is to refine the details in the optimized results that deviate from the ground truth by learning to integrate features from both high and low layers. The RRM consists of four stages each for the encoder and decoder. Each stage involves a convolution operation to extract image features. Each layer has a set of 64 3×3 convolutional filters to capture specific feature information. Batch normalization and ReLU activation functions are applied after each convolution. The bridge connection layer follows the same structure.

**Figure 8:**
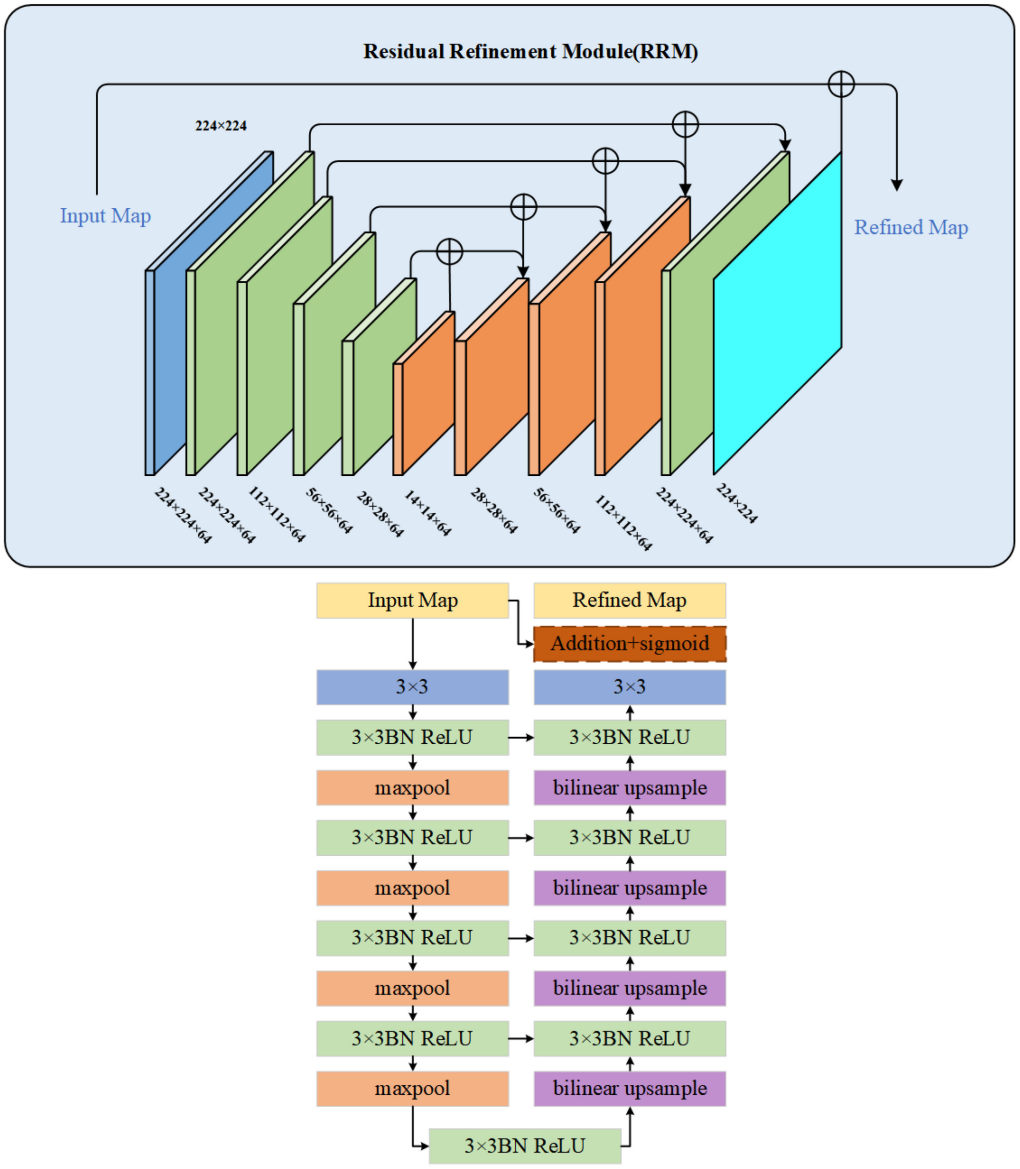
Structure diagram of RRM module.

Upon receiving the fused feature map from the original network’s decoder, the encoder utilizes non-overlapping max pooling for downsampling to preserve global texture information. The decoder employs up-sampling with bilinear interpolation to restore the fine features to the original size. Finally, the module outputs the result of the saliency feature map. This design enables the continuous capture of detailed information at different scales and enhances the completeness of boundary semantic features. The structure of RRM is depicted in **Figure 8**.

### 3.4 CT image segmentation method based on improved Deeplab V3+ network

**Figure 9:**
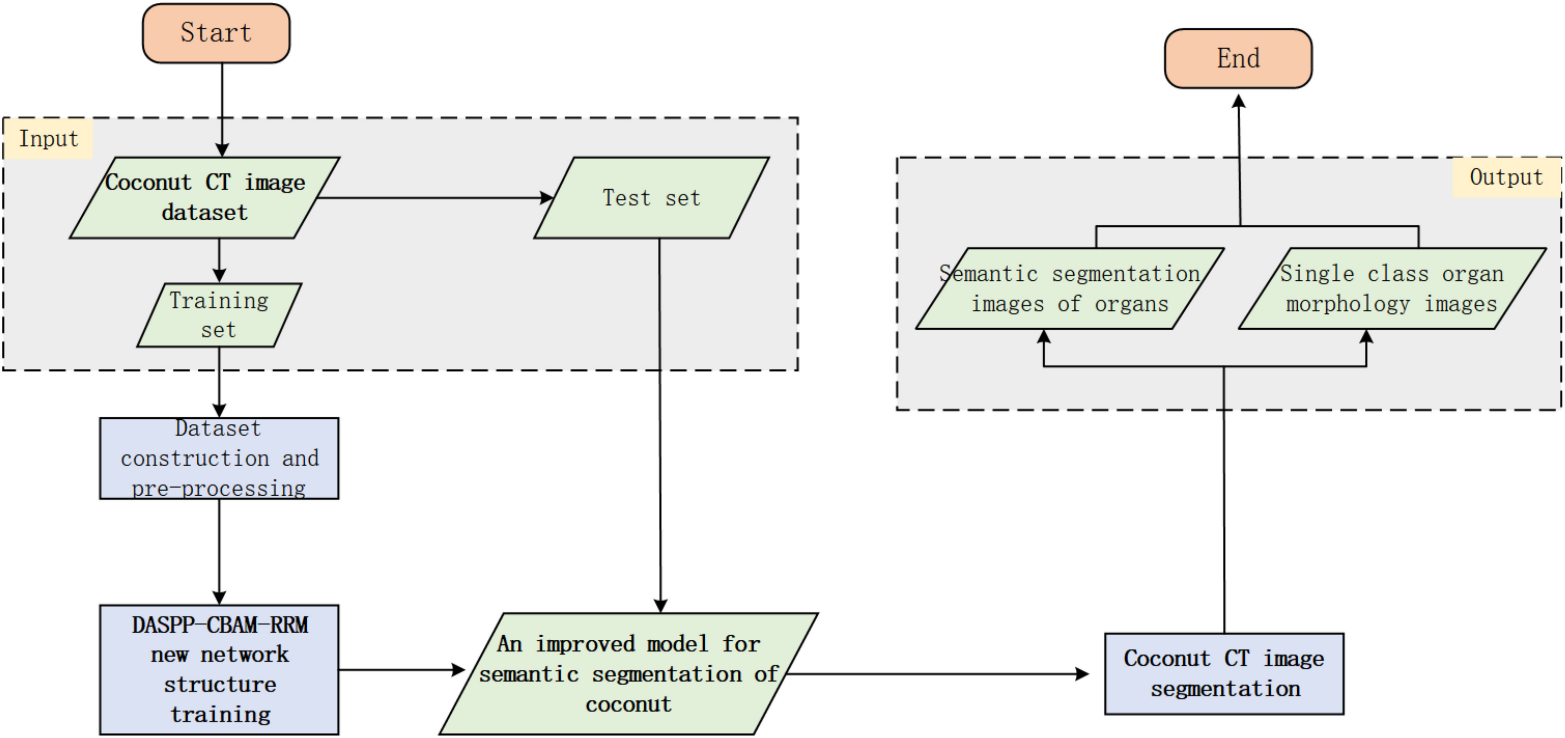
Flow chart of CT image segmentation based on improved Deeplab V3+ network.

After making improvements to the network model, and based on the established dataset, the two main components are integrated into the entire segmentation method. The logical flow of the process is designed as shown in **Figure 9**. The diamond boxes represent the results obtained before and after algorithm training and testing, while the rectangular boxes represent the operations during the training and testing process.

A self-built dataset of coconut CT images is used, including the original images and the corresponding ground truth segmentation images. The types and quantities of images can be selected and divided into training and testing sets as needed. For network model training, the original coconut CT images are used as inputs to the entire model, with the ground truth segmentation images as the supervision. The training process is end-to-end. After training, the improved Deeplab V3+ model for coconut CT image segmentation is obtained.

Then, in the segmentation stage, a given original coconut CT image from the testing set is used. With the trained improved segmentation model, specific target organs can be segmented from the image. If it is necessary to view a specific organ separately, the pixel color values can be traversed to detect and extract the target region. Since the CT scanner has a fixed scale set when generating slice images, the values obtained from the semantic image can be transformed according to the scale to obtain actual quantified data of the target organ. Subsequently, segmentation experiments and validations will be conducted using this method.

## 4 Experiment

### 4.1 Experimental environment

The CT images were captured using a dual-source CT scanner (Somatom Definition Flash, Siemens, Germany). Each time, the coconut was placed uniformly with the top facing upwards and the bottom placed on a fixed mold. They were sequenced according to the month of growth, and positions were marked with a marker on both the fixed mold and the coconut to ensure data uniformity and completeness throughout long-term scanning. The CT scan parameters were as follows: slice thickness/increment = 0.6mm/75%, tube voltage 120kV, tube current 250mAs, field of view (FOV) 400mm×400mm, gantry rotation speed 0.5s/rotation.

Model training was conducted on a Dell workstation with the Ubantu 20.04 operating system. It includes 24G of video memory, an RTX3090 graphics card, an Inter i7 CPU, and was developed on the Pycharm platform. The version of Pytorch used was torch1.10, with cuda version 11.4. The model was trained using our own constructed Coconut CT Imaging Dataset (CIDCO).Since the previously established coconut CT dataset was categorized and stored in separate folders according to coconut variety and growth stage, to ensure comprehensive training data, images were randomly selected from each category. Five categories were chosen for semantic segmentation: absorber, solid endosperm, liquid endosperm, embryo, and background. Due to the large differences in the internal organs of coconuts at different developmental stages, some organ categories were missing.Taking into account the prevention of an excessive number of images with the same stage and same features, in order to maintain a relatively balanced number of categories in the experimental dataset, the number of pictures containing various organs was adjusted flexibly. In the end, a total of 1470 images were confirmed as experimental data and were divided into a training set and a test set at a ratio of 8:2.

### 4.2 Training parameters and evaluation metrics

The improved semantic segmentation algorithm adopts a fully supervised learning approach during training. All methods are conducted on the same hardware. The hardware environment for this experiment consists of a workstation based on a 64-bit Ubuntu 20.04 operating system, Intel i7-1050H CPU, 16GB of RAM, 24G of video memory, and an NVIDIA GeForce GTX3090 graphics card. The software environment includes the Pytorch 1.1.0 framework, CUDA version 11.4, Python 3.6, and the Pycharm development platform. The input images are uniformly adjusted to a size of 256×256 pixels. The hyperparameters for the training of the coconut CT image segmentation model are as follows: The Adam optimizer is used with a learning rate of 0.0001, a training batch size of 4, momentum set to 9, a weight in the loss function of 0.7, and the loss function being a combination of Dice loss and focal loss. The total number of training epochs is set to 150.

To validate the effectiveness and robustness of the improved network model, we use IoU (Intersection over Union) and PA (Pixel Accuracy) to measure the segmentation results of individual organs. mIoU (mean Intersection over Union), mPA (mean Pixel Accuracy), and F1_score are used to evaluate the model’s overall semantic segmentation capability for coconut CT images. These are commonly used evaluation metrics in semantic segmentation tasks.IoU refers to the ratio of the intersection and union of the model’s prediction results and actual values for a single category of a coconut organ. PA refers to the proportion of correctly predicted pixels in a single organ category to the total number of pixels. mIoU represents the average of the ratios of intersections and unions of prediction results and actual values for each category of coconut organs. mPA is calculated by first computing the *PA* for each organ class of the coconut, and then taking the average of the *PA_s_* for all classes.F1_Score represents a comprehensive score for the correctness of the final results. Thus, the larger the value of these indicators, the better the segmentation effect of the model. Their calculation formulas are as per Equations 1–7, where *TP* represents the number of correct detections, *FP* is the number of false detections, *FN* is the number of undetected quantities, *k* represents the number of categories, *p_ii_* indicates the number of correctly classified pixels; *p_ij_* is the number of pixels of class *i* predicted as class *j*, Precision(*i*) represents the precision of class *i*, Recall(*i*) represents the recall rate of class *i*, and *r_i_* represents the proportion of the number of samples of class *i* in the total samples.


(Eq. 1) $$\text{ Precision }=\frac{TP}{TP+FP}$$

(Eq. 2) $$\text{Recall}=\text{Sensitivity}=TPR=\frac{TP}{TP+FN}$$

(Eq. 3) $$F1_{-}S\text{ core}=\frac{2^{*}\text{ Precision }*\text{ Recall}}{\text{Precision }+\text{ Recall}}$$

(Eq. 4) $$PA=\frac{\sum_{i=0}^{k}p_{ii}}{\sum_{i=0}^{k}\sum_{j=0}^{k}p_{ij}}$$

(Eq. 5) $$mPA=\frac{1}{k+1}\sum_{i=0}^{k}\frac{p_{ii}}{\sum_{j=0}^{k}p_{ij}}$$

(Eq. 6) $$IoU=\frac{\sum_{i=0}^{k}p_{ii}}{\sum_{i=0}^{k}\left(\sum_{j=0}^{k}p_{ji}+\sum_{j=0}^{k}p_{ij}p_{ii}\right)}$$

(Eq. 7) $$mIoU=\frac{1}{k+1}\sum_{i=0}^{k}\frac{p_{ii}}{\sum_{j=0}^{k}p_{ij}+\sum_{j=0}^{k}p_{ji}-p_{ii}}$$

### 4.3 Ablation study and model comparison

#### 4.3.1 Module ablation study

To verify the effectiveness of our proposed improvements, we designed an ablation study in which we run the model on the same dataset, subtracting one of the three modules from the improved model. ‘All’ represents the complete modules that we have added. The training process uses the same parameter configuration, and the final results are shown in **Table 1**.

**Table 1:** Module ablation data table.

Keep the module	Background		Solid Endosperm		Embryo	Haustorium		Liquid Endosperm		mIoU	mPA	F1_Score
	IoU	PA	IoU	PA	IoU PA	IoU	PA	IoU	PA			
**DASPP+CBAM**	0.99	0.99	0.82	0.92	0.74 0.85	0.84	0.88	0.71	0.93	82.46	91.86	90.09
**RRM+CBAM**	0.99	0.99	0.82	0.92	0.75 0.85	0.85	0.90	0.72	0.91	82.99	92.02	90.43
**DASPP+RRM**	0.99	0.99	0.82	0.92	0.67 0.72	0.85	0.90	0.62	0.94	79.43	89.98	87.93
**ALL(D+C+R)**	0.99	0.99	0.82	0.93	0.75 0.85	0.84	0.89	0.72	0.92	83.10	92.05	90.50

According to the data in the table, the network structure improved by the three modules shows the best overall performance. When focusing on individual organs, the improved new network has a higher pixel accuracy than the other comparative modules. When faced with complete organ images showing different features, the model’s mIoU, mPA, and F1_Score all outperform structures missing a module. For the task of semantic segmentation of coconut organs, focusing on the entire target area’s features and supplementing with local boundary information is the optimal solution. Thus, it is confirmed that this point of improvement can significantly enhance the robustness and accuracy of the segmentation method.

#### 4.3.2 Comparison of segmentation results from different models

**Figure 10:**
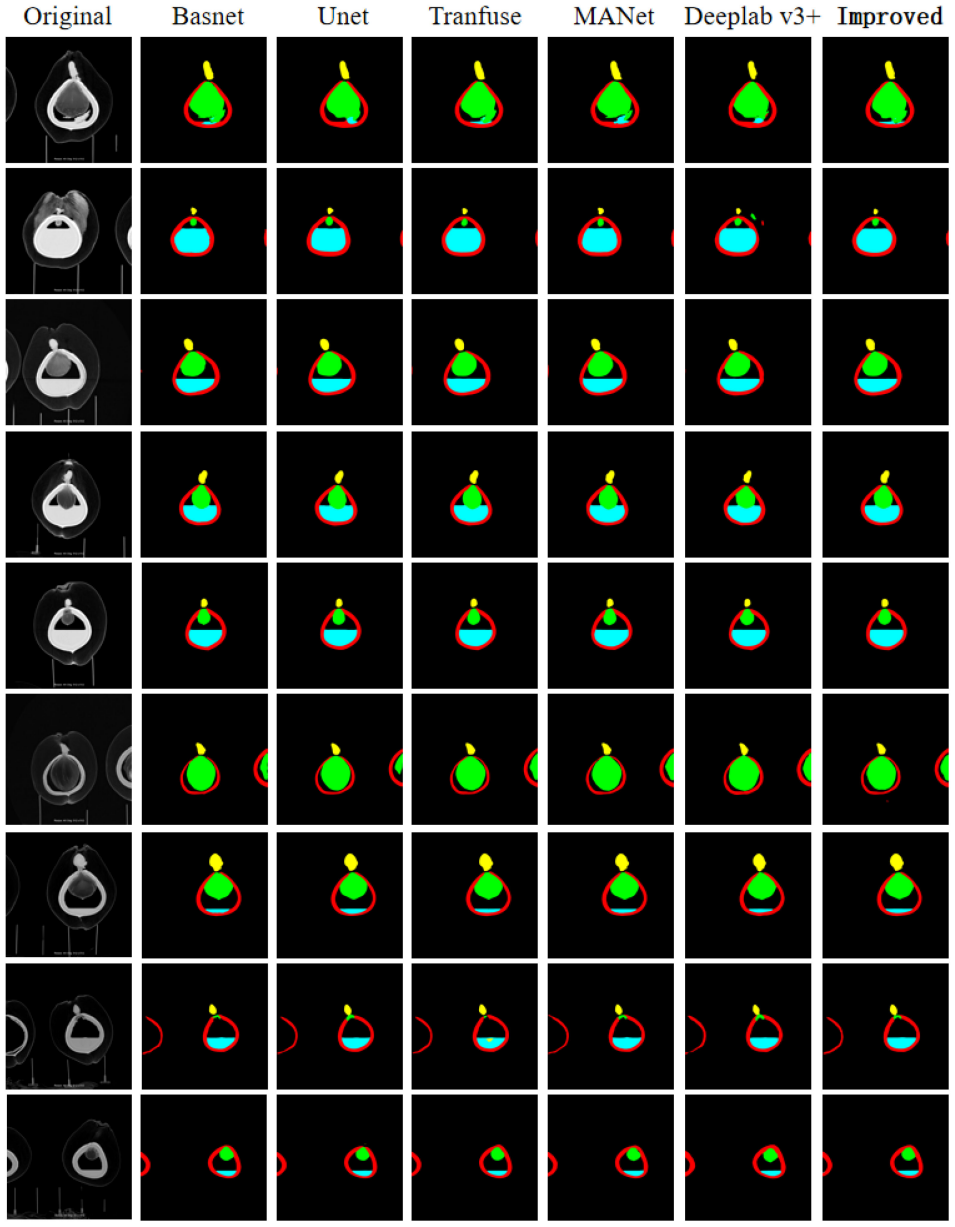
Semantic segmentation effect of different models.

In the same dataset, we compare our proposed model with commonly used segmentation models to verify our model’s excellent segmentation capability. We selected five models, namely Basnet, Unet, Transfuse, MANet, and Deeplab v3+, using IoU, PA, mIoU, mPA, and F1_Score as evaluation metrics. We compare and analyze the results from both qualitative and quantitative perspectives, as shown in **Figure 10** and **Table 2**.

**Table 2:** Model comparison table.

Network model	Background		Solid Endosperm		Embryo		Haustorium		Liquid Endosperm		mIoU	mPA	F1_Score
	IoU	PA	IoU	PA	IoU	PA	IoU	PA	IoU	PA			
**Basnet**	0.99	0.99	0.84	0.93	0.39	0.41	0.84	0.89	0.48	0.93	71.30	83.69	81.00
**Unet**	0.99	0.99	0.82	0.91	0.46	0.50	0.85	0.89	0.51	0.91	72.80	84.57	82.55
**Tranfuse**	0.99	0.99	0.83	0.92	0.46	0.54	0.84	0.89	0.55	0.88	73.94	84.92	83.49
**MANet**	0.99	0.99	0.83	0.92	0.65	0.76	0.85	0.90	0.74	0.94	81.75	90.82	89.54
**Deeplab v3+**	0.99	0.99	0.79	0.92	0.65	0.70	0.84	0.90	0.64	0.88	78.47	88.34	87.36
**Improved model**	0.99	0.99	0.82	0.93	0.75	0.85	0.84	0.89	0.72	0.92	83.10	92.05	90.50

From **Table 2**, it is clear that the improved model performs better than the majority of models in terms of Intersection over Union (IoU) and Pixel Accuracy (PA) when facing segmentation of individual organ classes. This is especially apparent for liquid endosperm and embryos. Other models are only comparable to the improved model in one or two data points. For the semantic segmentation of the entire image, the improved model has a clear advantage in terms of mean Intersection over Union (mIoU), mean Pixel Accuracy (mPA), and F1_Score. These three metrics show that the values have improved compared to the comparison models, proving the effectiveness of the improvement method proposed in this chapter. Apart from quantitative results, **Figure 10** shows the segmentation effects of each model at the image level, demonstrating that the improved model still has a higher accuracy in segmentation at a qualitative level.

### 4.4 Organ extraction

**Figure 11:**
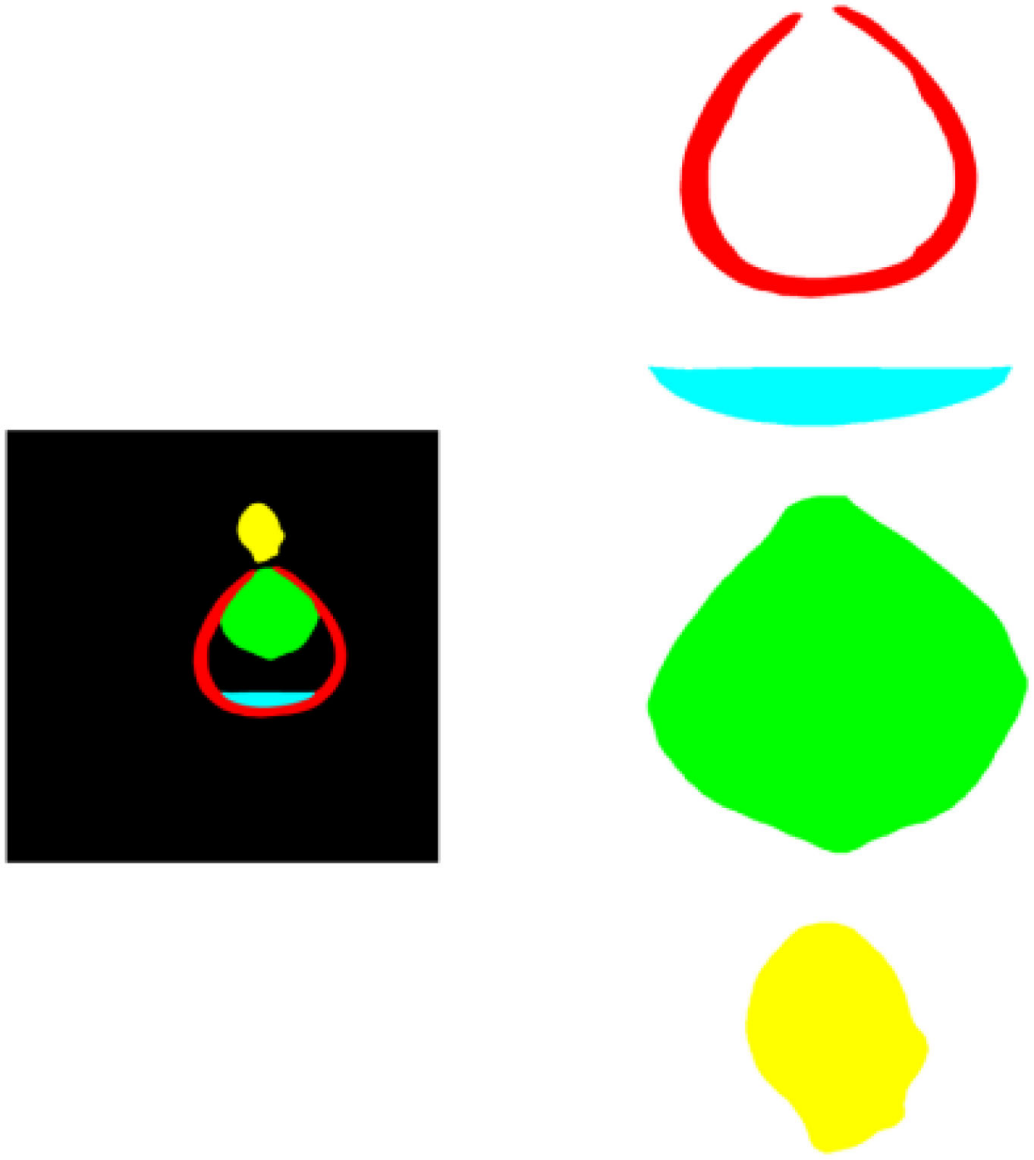
Example of single class organ extraction.

Considering that in actual scenarios, it may be necessary to extract a particular organ for analysis, we set up an organ extraction and data quantification section. After inputting the images to be operated on into the model, we obtain the semantic images of coconuts. We then create a corresponding number of blank images of the same size, traverse all pixels in the semantic image, and follow the principle of point-to-point correspondence in the target organ based on the RGB value in the semantic image to make the corresponding points in the blank image the same value. This way, we can obtain the image of the target organ alone. In terms of determining the growth and development quality of the coconut, quantitative data of the organs is one of the reference pieces of information, in addition to making judgements in the form of two-dimensional images. Whether it’s the complete semantic image of the coconut or a particular organ that has been extracted, data can still be obtained through the RGB value of the pixel points. For example, the height of the embryo can be determined because, in the semantic image, the embryo is characterized by the color green. One can start from the top of the image and gradually traverse downwards in the form of a horizontal line. When the RGB value of a pixel point becomes (0, 255, 0), it is marked as point A. Then, using the same method, traverse from the bottom of the image upwards, and when you encounter a pixel point with the same value, mark it as point B. The distance between points A and B is the height of the embryo. When dealing with an embryo with a significant curvature, it can be rotated to be relatively parallel to the y-axis, and then the point traversal method can be used. **Figure 11** shows an example of the extracted image results.

## 5 Conclusion and prospects

This chapter starts from the perspective of the black box phenomenon present in the development process of the coconut fruit. We used CT non-destructive observation to acquire images of coconuts at various stages and of various varieties, thus establishing a CT image dataset for coconuts. This work fills the gap in image resources for coconuts. On this basis, we addressed the issue of traditional semantic segmentation models not performing well on coconut CT images. We replaced the original Atrous Spatial Pyramid Pooling (ASPP) block with a Dense Atrous Spatial Pyramid Pooling (DASPP) module, resolving information loss due to sparse sampling. Then, we added the Convolutional Block Attention Module (CBAM) to the network, enabling it to better capture the features of coconut organs and reduce the interference of irrelevant redundant information. Finally, a residual refinement module was embedded after the decoder to enhance the boundary information between closely connected organs. This allows the network to acquire richer global feature information and optimize boundary details, thereby improving the semantic segmentation accuracy of coconut CT images. During the model training process, we used multi-state feature coconut images to improve the model’s robustness. Finally, detailed model comparisons and ablation experiments were carried out. The results of the evaluation indicators and the semantic segmentation effect images both quantitatively and qualitatively demonstrate the improved model’s high-precision segmentation ability on coconut CT images. Furthermore, individual organ morphology and quantitative data can be obtained from the semantic segmentation images to increase reference information during the development process of the coconut. This is beneficial in assisting decision-makers to make scientific judgments on the development status and growth stage of the coconut.

In our future research work, we will analyze the high-precision organ morphology and quantitative data obtained from the segmentation model to further mine the laws of coconut growth and development. At the same time, we will incorporate image morphology changes to construct a visualized standard development process for the coconut, thereby making more precise predictions of coconut intelligent development. Furthermore, we aim to deploy our model on mobile devices to provide more reference information and decision support for optimizing coconut breeding. This will aid coconut cultivators in better managing their cultivation practices, with the goal of achieving and continuously surpassing targets for high yield and high-quality coconuts.

## Data availability statement

The raw data supporting the conclusions of this article will be made available by the authors, without undue reservation.

## Author contributions

QL was in charge of building the modified Deeplab V3+ model and writing the paper, YZ was in charge of the model comparison and ablation experiments. JC and CS were in charge of building the data set, JC was in charge of scanning the CT images, CS was in charge of providing all kinds of coconut. MH was in charge of the coordination of the whole workflow and thesis guidance. MC and CL were in charge of the image tagging. SL was responsible for assisting JC to perform CT scans and record data. All authors contributed to the article and approved the submitted version.
